# Identification of a major QTL, *Parth6.1* associated with parthenocarpic fruit development in slicing cucumber genotype, Pusa Parthenocarpic Cucumber-6

**DOI:** 10.3389/fpls.2022.1064556

**Published:** 2022-12-14

**Authors:** Shilpa Devi, Parva Kumar Sharma, Tusar Kanti Behera, Sarika Jaiswal, G. Boopalakrishnan, Khushboo Kumari, Neha Kumari Mandal, Mir Asif Iquebal, S. Gopala Krishnan, Bharti, Chandrika Ghosal, Anilabha Das Munshi, Shyam Sundar Dey

**Affiliations:** ^1^ Division of Vegetable Science, ICAR-Indian Agricultural Research Institute, New Delhi, India; ^2^ Centre for Agricultural Bioinformatics, ICAR-Indian Agricultural Statistics Research Institute, New Delhi, India; ^3^ ICAR-Indian Institute of Vegetable Research, Varanasi, India; ^4^ Division of Genetics, ICAR-Indian Agricultural Research Institute, New Delhi, India; ^5^ Division of Sample Survey, ICAR-Indian Agricultural Statistics Research Institute, New Delhi, India

**Keywords:** cucumber, parthenocarpy, inheritance, QTL-seq, molecular mapping, candidate genes

## Abstract

Parthenocarpy is an extremely important trait that revolutionized the worldwide cultivation of cucumber under protected conditions. Pusa Parthenocarpic Cucumber-6 (PPC-6) is one of the important commercially cultivated varieties under protected conditions in India. Understanding the genetics of parthenocarpy, molecular mapping and the development of molecular markers closely associated with the trait will facilitate the introgression of parthenocarpic traits into non-conventional germplasm and elite varieties. The F_1_, F_2_ and back-crosses progenies with a non-parthenocarpic genotype, Pusa Uday indicated a single incomplete dominant gene controlling parthenocarpy in PPC-6. QTL-seq comprising of the early parthenocarpy and non-parthenocarpic bulks along with the parental lines identified two major genomic regions, one each in chromosome 3 and chromosome 6 spanning over a region of 2.7 Mb and 7.8 Mb, respectively. Conventional mapping using F_2:3_ population also identified two QTLs, *Parth6.1* and *Parth6.2* in chromosome 6 which indicated the presence of a major effect QTL in chromosome 6 determining parthenocarpy in PPC-6. The flanking markers, SSR01148 and SSR 01012 for *Parth6.1* locus and SSR10476 and SSR 19174 for *Parth6.2* locus were identified and can be used for introgression of parthenocarpy through the marker-assisted back-crossing programme. Functional annotation of the QTL-region identified two major genes, *Csa_6G396640* and *Csa_6G405890* designated as probable indole-3-pyruvate monooxygenase YUCCA11 and Auxin response factor 16, respectively associated with auxin biosynthesis as potential candidate genes. *Csa_6G396640* showed only one insertion at position 2179 in the non-parthenocarpic parent. In the case of *Csa_6G405890*, more variations were observed between the two parents in the form of SNPs and InDels. The study provides insight about genomic regions, closely associated markers and possible candidate genes associated with parthenocarpy in PPC-6 which will be instrumental for functional genomics study and better understanding of parthenocarpy in cucumber.

## Introduction

Cucumber (*Cucumis sativus* L.) is grown commercially in tropical and subtropical climates around the world (Pradeepkumara et al., 2022). In the Indian sub-continent, cucumber is grown from the highlands to the plains under open fields and protected conditions, including riverbeds. India is considered as the home of cucumber and has a wide range of genetic diversity and variation depending on growth habits, fruit size, fruit composition, and skin color besides several other agronomically important traits (Staub et al., 1997), but this variation has never been fully utilized in the crop improvement. The cultivated cucumber has a narrow genetic base with only 3-8% polymorphism within the cultivated genotypes, and 10-25% between plant species (Behera et al., 2011). The small, diploid genome (367 Mb), annual growth pattern, autogamous mating system, and relatively short life cycle (~ 3 months from generation to offspring) provide important genetic benefits (Wang et al., 2020) and detailed genomics-based studies in cucumber.

In most of the Angiosperms, fruit formation usually occurs after successful pollination followed by fertilization of eggs, which results in ovary growth, however, fruit development without pollination and fertilization is referred to as parthenocarpy (Li et al., 2014). Parthenocarpic fruits are seedless as ovules fertilization is disrupted due to changes in the basic genetic makeup involved in fertilization processes. Sources of genetic parthenocarpy are either obligate or facultative by nature. In sexually transmitted species, parthenocarpic genotypes need to be facultative in nature for successful development of fruits when pollinated. Alternatively, the obligate parthenocarpy can be found in asexually propagated plants (Gorguet et al., 2005). From a consumer perspective, parthenocarpy is a possible way to improve fruit quality and total productivity in several fruit crops. If seed setting fails, flower mortality is a common way to avoid wasting of resources. However, parthenocarpic genotypes are also found in wild or non-fruit species, indicating that there may be a variety of factors that cause the formation of seedless fruit in higher plants. The viability and permanence of parthenocarpy in a variety of plants is mainly the result of human selection (Varoquaux et al., 2000). Parthenocarpy in cucumber is determined by a complex interaction between several genetic factors and phytohormones (Sharif et al., 2022; Gou et al., 2022). Various phytohormones, especially gibberellins, cytokinins and auxins are involved in the processes that follow pollination and fertilization and these are essential factors for fruit and seed development (Fos et al., 2001). Growing seeds are major source of phytohormones that stimulate fruit growth and development (Ozga et al., 2002). The use of gynoecy in combination with parthenocarpy is necessary as cucumber exhibits facultative parthenocarpy as seeded fruit set can occur in parthenocarpic varieties when fertilised with viable pollen source. Gynoecious varieties are advantageous because of increased numbers of pistillate flowers, and thus greater opportunities for higher fruit set and per unit production. Parthenocarpic cucumber varieties offer several advantages over conventional seeded varieties. Parthenocarpic varieties are able to set fruits sequentially without suffering from first-fruit inhibition (Denna, 1973; Sun et al., 2006a). Parthenocarpy should be combined with stable gynoecious habit, because the fruits formed after fertilization of parthenocarpic plants become misshapen, have no economic value and lead to loss of productivity in case the female flowers received viable pollen. Selection of diverse genotypes to be used as a parent in the development of the F_1_ hybrid to achieve higher yield, uniformity and suitability for protected cultivation (2016; 2017; Jat et al., 2015) is necessary in crop improvement programme. Therefore, the development of molecular markers closely associated with parthenocarpy and its marker-assisted introgression into diverse back-grounds is necessary to facilitate hybrid breeding programme.

Marker assisted selection (MAS) can enhance the efficiency of traditional breeding. In cucumber, breeding of parthenocarpic lines based on molecular markers provides a faster and more efficient way as selection can be based on genotypes itself rather than the phenotypes. There are two key requirements in successful of MAS, *i.e.* markers should be closely linked to target genes and a moderately saturated or high density genetic linkage map (Miao et al., 2011). The development of genetic linkage maps in cucumber have made possible for molecular characterization of important economic traits which includes fruit quality (Wenzel et al., 1995), resistance to diseases (Park et al., 2000; Zhang et al., 2010), yield (Serquen et al., 1997b; Fazio et al., 2003a), gynoecious sex and fruit colour (Miao et al., 2011) and yellow fruit flesh (Lu et al., 2015). Genetic studies have been largely inconsistent on the mode of inheritance for parthenocarpy in cucumber and have ranged from proposals of a single gene to complex multigenic inheritance (Pike and Peterson, 1969; De Ponti and Garretsen, 1976; El-Shawaf and Baker, 1981; Kim et al., 1992b). In the past, parthenocarpy has been studied by several workers to unravel the genetic and physiological basis of this extremely important trait (Fu et al., 2008; Li et al., 2014; Su et al., 2021; Gou et al., 2022; Mandal et al., 2022). The parthenocarpic genotypes of cucumber can set fruit without pollination however normal seed formation happens with successful pollination with viable pollen grains. This typical phenomenon in cucumber is attributed to the facultative parthenocarpic nature. The majority of the studies in the last two decades suggested that multiple QTLs across the genome are responsible for parthenocarpic fruit development in cucumbers. In a European greenhouse-slicing cucumber genotype, EC-1 parthenocarpy was found to be determined by one major and stable QTL in chromosome 2 (*Parth 2.1*) revealed through a F_2:3_ population (Wu et al., 2015). In north American pickling type cucumber 2A, seven QTL were detected for parthenocarpy and one QTL each on chromosomes 5 and 7 (*parth5.1* and *parth7*.*1*) and two on chromosome 6 (*parth6.1* and *parth6.2*) were found govern parthenocarpy (Lietzow et al., 2016). Besides, in a south China ecotype cucumber, 4 novel QTLs associated with parthenocarpy were detected (Niu et al., 2020).

There is a broad consensus based on the available reports that parthenocarpic fruit set is complex in nature and genomic regions in different chromosomes are responsible for induction of parthenocarpic fruit development in cucumbers. The present study was conducted to identify and map the genomic regions associated with parthenocarpy in one of the commercially cultivated gynoecious parthenocarpic genotype, Pusa Parthenocarpic Cucumber-6 (PPC-6) through QTL-seq approach. Identification of closely linked PCR-based markers and possible identification of candidate genes associated with parthenocarpy in PPC-6 would facilitate the marker-assisted back-cross breeding and characterization of parthenocarpic trait in cucumber.

## Materials and methods

### Plant materials

The commercially cultivated parthenocarpic genotype, PPC-6 is cultivated widely in India under protected condition and was used as one of the parents for studying the inheritance and development of mapping population for parthenocarpic trait. In contrast, the non-parthenocarpic parent, Pusa Uday (PU), an Indian type cultivar suitable for cultivation under open field conditions was taken for the study. The F_1_ progeny was developed by crossing the PU with PPC-6 under protected conditions. Development of F_1_, F_2_ and back-cross progenies were undertaken under protected conditions. The plants of inbreds and developed progenies were grown under protected conditions using the standard agronomic practices developed by the Division of Vegetable Science, ICAR-Indian Agricultural Research Institute, New Delhi.

### Inheritance of parthenocarpy

The parthenocarpic line, PPC-6 was crossed with the non-parthenocarpic cultivar, Pusa Uday (PU). The resulting F_1_ generation was selfed to obtain a sufficient number of F_2_ population. The female flowers were covered with a butter paper bag, one day prior to anthesis to avoid cross-pollination, and pollens collected from the freshly opened male flowers were used for pollination. The observations were recorded for development of parthenocarpic fruits up to the 20^th^ node. If a plant produced parthenocarpic fruits up to the 1^st^ to 5^th^ nodes, it was considered an early parthenocarpic plant while if the fruit set occurred beyond the 10^th^ node, plants were categorized as late parthenocarpic. A total of 498 F_2_ progenies were grown for recording observation on parthenocarpy and observation was recorded from 400 plants. The observations were recorded separately for early parthenocarpy, late parthenocarpy, and non-parthenocarpy. The goodness of fit of the observed values to the expected segregation ratio for parthenocarpic and non-parthenocarpic plants was tested using the classical Chi-square (χ^2^) test as expressed below (Panse and Sukhatme, 1985):


$$\chi^{2}=\frac{{\left(Observed-Expected\right)}^{2}}{Expected}$$

### DNA extraction and whole genome resequencing

Approximately 15g of leaf samples were collected for DNA isolation from 30-35 day-old seedlings at the active vegetative stage during early morning hours. The collected leaf samples were packed in aluminum foil and labeled properly, then frozen into liquid nitrogen and stored at -80°C for further use. Total DNA was isolated from the individual parental lines, F_1_ hybrids, and mapping populations using the modified cetyl trimethyl ammonium bromide (CTAB) method (Saghai-Maroof et al., 1984). The genomic DNA samples were adjusted to 50ng DNA/µl and stored at 4°C until used as the templates for PCR amplification and sampling for sequencing. The quality and quantity of the extracted DNA were estimated with an Eppendrof Biospectrometer confirmed by running on 0.8% w/v agarose gel.

### QTL-seq for identification of genomic regions associated with parthenocarpy

**Figure 1:**
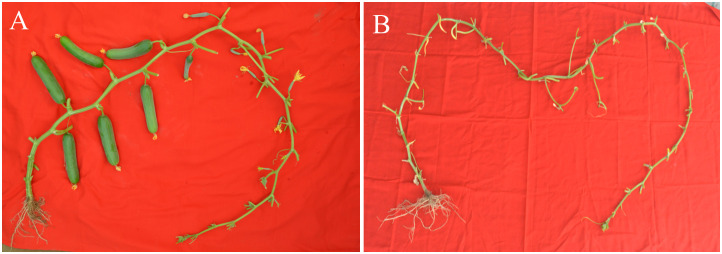
Fruit set in the contrasting parental lines **(A)** Pusa Parthenocarpic Cucumber-6 with parthenocarpic fruit development **(B)** Pusa Uday with no parthenocarpic fruit set under protected condition.

For QTL-seq analysis, 498 F_2_ progenies derived from the crossing of the PU × PPC-6, were grown under polyhouse with a partially controlled environment along with their parents. Observation on parthenocarpy was recorded 45 days after sowing when the plants were in the full reproductive stage. One plant from each of the parents, PU and PPC-6 along with two extreme bulks constituting twenty plants each from the parthenocarpic and non-parthenocarpic types were used for sampling (**Figure 1**). Young leaves from each selected plant were used for the isolation of genomic DNA. After DNA isolation and purification, quantification was done using a Qubit (Thermofisher Scientific, USA). An equal quantity of DNA from each plant taken for bulking to constitute the final bulks.

### Pre-processing of reads

Paired-end Illumina reads were obtained for both the parents and the bulks in duplicates. All the reads were 2*150 in length. FastQC version 0.11.8 (Andrews, 2010) was used for visualization of various read parameters and the presence of low-quality bases and of Illumina adapters. Based on the FastQC report the reads were cleaned and trimmed using Trimmomatic v0.39 (Bolger et al., 2014) with command parameters ‘ILLUMINACLIP:TruSeq3-PE.fa:2:30:10:2:keepBothReads SLIDINGWINDOW:4:15 MINLEN:50’. Here, TruSeq3-PE.fa is a fasta file containing Illumina adapter sequences. The obtained high-quality reads were used further for the identification of QTL regions.

### QTL-Seq analysis

**Figure 2:**
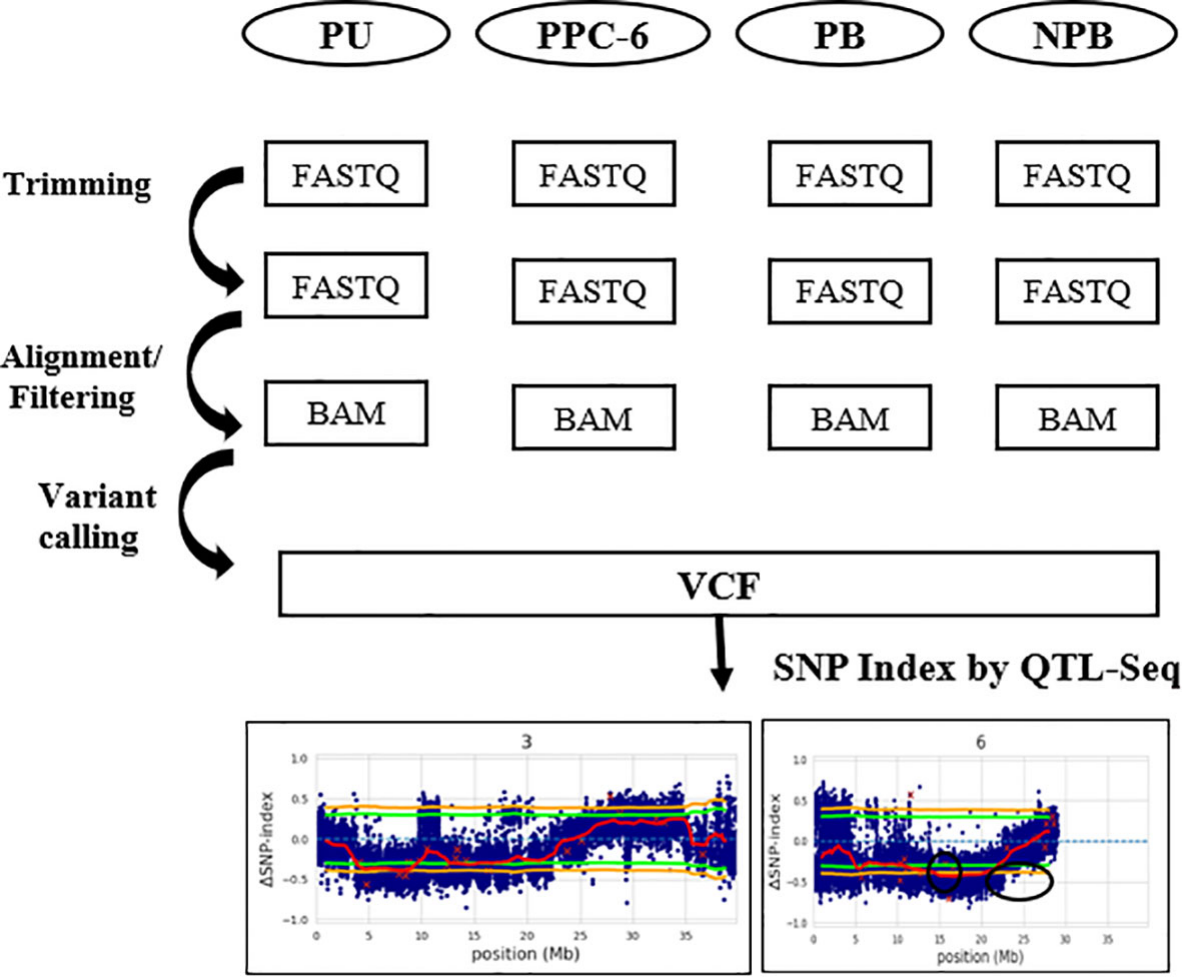
Schematic representation of the pipeline used for the QTL- Seq analysis.

QTL-Seq is a fast and efficient method to identify loci related to agronomically important traits (Takagi et al., 2013) in plants. Bulked Segregation Analysis (BSA) is the root of QTL-Seq that detects genomic location(s) showing significant variations in contrasting parents and progenies, produced by the contrasting parents. BSA relies on two main parameters, *namely*, SNP-index and Δ (SNP-index) (Abe et al., 2012; Takagi et al., 2013). SNP-index is the ratio of a number of reads having a variation, to the total number of reads at a particular position. While Δ (SNP-index) is the difference in the SNP indices of contrasting bulks. The range of SNP index varies from 0 to 1 depending on the parent chosen as a reference. For instance, if parent 1 is used as a reference, and the reads aligned at a particular locus do not have any variation this means that all the loci have been contributed from parent 1 and hence SNP-index = 0, but if all the reads aligned at a particular locus show variation, then it means that the loci have been contributed by another parent and therefore SNP-index = 1. For calculating the SNP-index, a sliding window approach was used with window size of 2000 kb and 100 kb increment followed by their averaging. Graphs were plotted for the Δ (SNP-index) against the chromosomal location. A Δ (SNP-index) value close to zero, indicates that no significant QTL is present at that locus for the studied trait. To obtain significant results, statistical confidence intervals of Δ (SNP-index) were also plotted for all SNP positions with read depth assuming that there are no QTLs as null hypothesis at 95% level of significance (Takagi et al., 2013). We used QTL-seq version 2.2.2 for the detection of significant QTL regions for parthenocarpy in cucumber (Sugihara et al., 2020). The parameters used were [-n1 20 -n2 20 -o qtlseq_results -F 2 -e *Cucumis sativus*] where n1 and n2 are the numbers of individuals in each bulk, -o is the output directory, F is the filial population (here we had F_2_) and -e is the dataset of reference genome for identification of the effects due to SNPs. All the other parameters were kept as default. This software uses BWA for the read alignment, SAMtools for filtering and BCF tools for variant calling (**Figure 2**).

### Conventional mapping using F_2:3_ progenies

**Figure 3:**
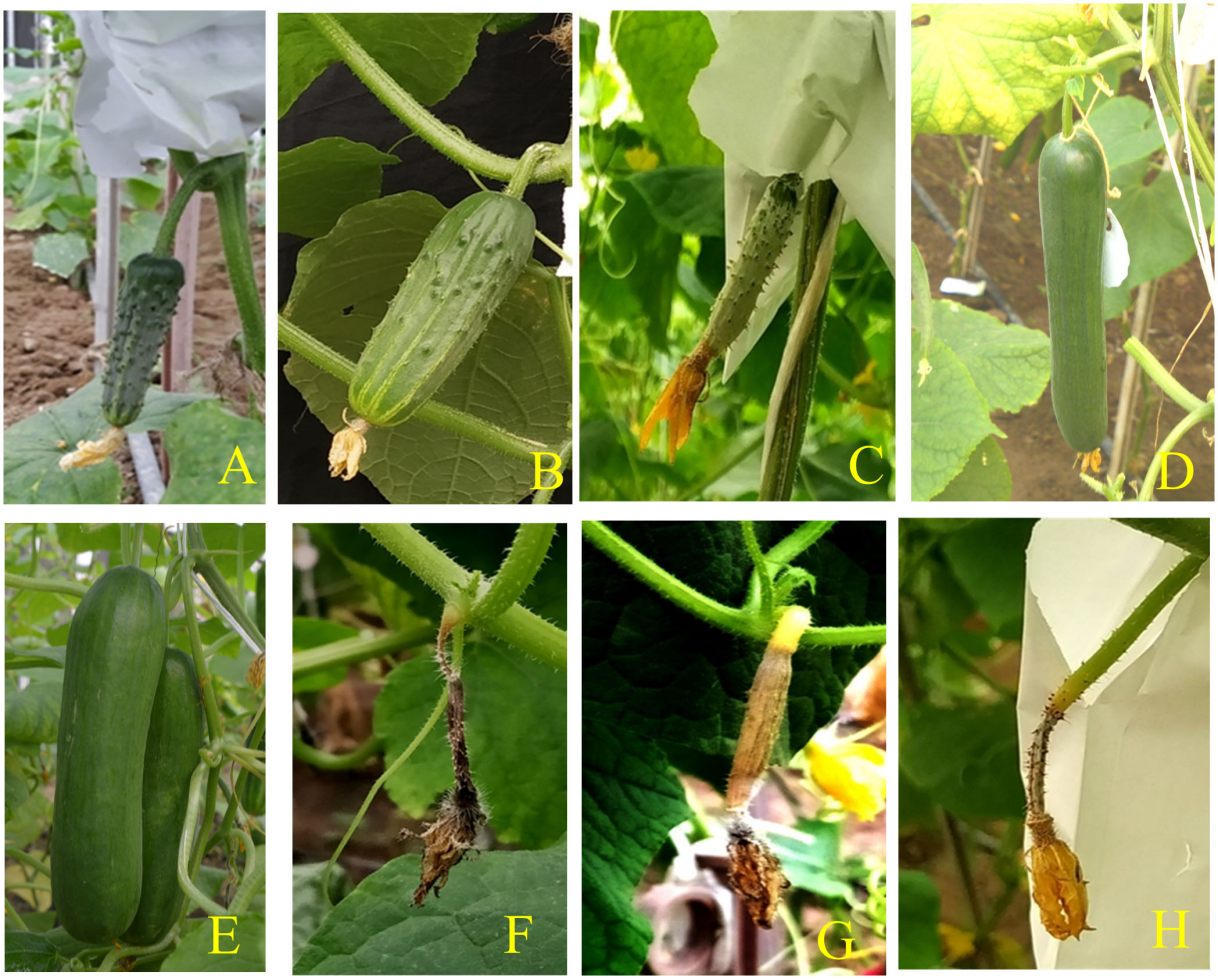
The pattern of fruit set in the segregating population at 10 days after anthesis **(A-H)**.

Phenotyping was conducted in F_2:3_ population developed through selfing of the individual F_2_ progenies for conventional molecular mapping of the parthenocarpy. An single F_1_ plant was selfed to obtain the F_2_ population. An F_2:3_ population comprising of 94 progenies derived from the cross between parthenocarpic and non-parthenocarpic parents was used to construct linkage map of cucumber. The F_2:3_ progeny rows along with parental lines were raised under an insect-proof net house during Kharif, July-October, 2021. The F_2:3_ progenies were grown in two replications with 10 plants in each replication for recording the observation on parthenocarpy. Eight female flowers were bagged one day prior to anthesis from the fifth node onwards on the main stem and eight more from the laterals. At 10 days after anthesis, well-developed and malformed fruits were counted as parthenocarpic fruit, whereas aborted ones were recorded as non-parthenocarpic (**Figure 3**) as suggested by Wu et al. (2016).

Genotyping was done using a large set of PCR-based markers uniformly distributed all throughout the cucumber genome. For the parental polymorphic survey, 1285 SSRs, Indels, CAPS markers were selected from the *Cucumis sativus* genome representing 7 linkage groups. In the present experiment, previously reported markers were used for the polymorphic study between the two genotypes, PPC-6 and PU (Miao et al., 2011; Zhu et al., 2016). Linkage analysis was performed using identified 123 polymorphic SSRs, Indels, CAPS markers for the construction of linkage map by IciMapping 4.1.0.0 at LOD threshold of 3.0 (Lander et al., 1987). Segregation of 123 SSRs, Indels, CAPS markers, and parthenocarpy was analyzed and genetic distance between markers was calculated using the Haldane and Kosambi map function (Kosambi, 1944). Parthenocarpy locus was mapped using Inclusive Composite Interval Mapping (ICIM) in ICIMapping 4.1.0.0 software (Wang et al., 2016).

## Results

### Inheritance of parthenocarpy

In the present study, the inheritance pattern of parthenocarpy was studied based on the classical dominant-recessive Mendelian model by grouping the cucumber plants into three categories of their fruit development i.e. early parthenocarpic, late parthenocarpic, and non-parthenocarpic fruit development. This information would facilitate the adoption of appropriate breeding strategies for the development of stable parthenocarpic cucumber lines and will improve the efficiency of selection procedures. The genotype, PU produced non-parthenocarpic fruits and it was considered to be homozygous for non-parthenocarpic fruit development. The development of parthenocarpic fruits in PPC-6 is characterised by early parthenocarpy with fruit setting from the beginning or from the base of the plant. Therefore, PPC-6 was used as a homozygous genotype for parthenocarpic fruit development. The F_1_ hybrid derived from the cross of PU × PPC-6 with heterozygous conditions produced some parthenocarpic fruits on the lower nodes*, i.e.* 10^th^ node and above (**Supplementary Figure 1**). In segregating F_2_ individuals, early, late and non-parthenocarpic fruits were recorded. Out of 400 plants, 307 produced either early parthenocarpic or late parthenocarpic fruits and 93 plants were recorded as non-parthenocarpic plants. The χ2 value indicated a good fit for segregation of parthenocarpy (early, late and non-parthenocarpy) in the F_2_ population populations confirmed with the expected ratio of 1:2:1 for early parthenocarpy, later parthenocarpy and non-parthenocarpy, respectively (**Table 1**). In the back cross progeny with the non-parthenocarpic genotype, PU the segregation for late parthenocarpy and early parthenocarpy were in the ratio of 1:1. Similarly, segregation of the plants for early parthenocarpy and late parthenocarpy was in the ratio of 1:1 for early and late parthenocarpy in the back-cross progenies with the parthenocarpic parent (**Table 2**).

**Table 1:** Evaluation of the parents along with F_1_ and F_2_ and back-cross progenies for studying the inheritance of parthenocarpy.

Crosses / Parents	Total number of plants	EP	LP	NP	Expected Ratio	χ^2^ -value	P-Value
PU	10	0	0	10	–	–	–
PPC-6	10	10	0	0	–	–	–
PU × PPC-6 (F_1_)	10	0	10	0	–	–	–
PU × PPC-6 (F_2_)	400	87	220	93	1:2:1	4.18	0.12
(PU × PPC-6) × PU	60	0	26	34	1:1	1.06	0.31
(PU × PPC-6) × PPC-6	60	27	33	0	1:1	0.60	0.43

EP, Early parthenocarpy; LP, late parthenocarpy; NP, Non-parthenocarpy.

**Table 2:** Summary of reads’ statistics of the re-sequenced samples of the parents along with the contrasting bulks.

Sample	Input Read Pairs	Clean reads	% Clean reads
PU (NPP)	43632883	41383222	94.84412
PPC-6 (PP)	43799473	41255039	94.19072
Parthenocarpic bulk (Bulk 1)	105030659	99904977	95.11982
Non-parthenocarpic bulk (Bulk 2)	107216823	103354754	96.39789

NPP, non-parthenocarpic parent; PP, parthenocarpic parent.

### Pre-processing of reads

The raw reads for both the parents and bulks were subjected to quality check and removal of adapter sequences. After pre-processing, the non-parthenocarpic parent (NPP), PU retained 41383222 clean reads while parthenocarpic parent (PP), PCC-6 retained 41255039 clean reads. In case of both the bulks, *i.e.*, Parthenocarpic bulk (PB) and Non-Parthenocarpic bulk (NPB), 99904977 and 103354754 cleans reads were obtained, respectively (**Table 1**).

### Identification of candidate genes in the QTL regions

**Figure 4:**
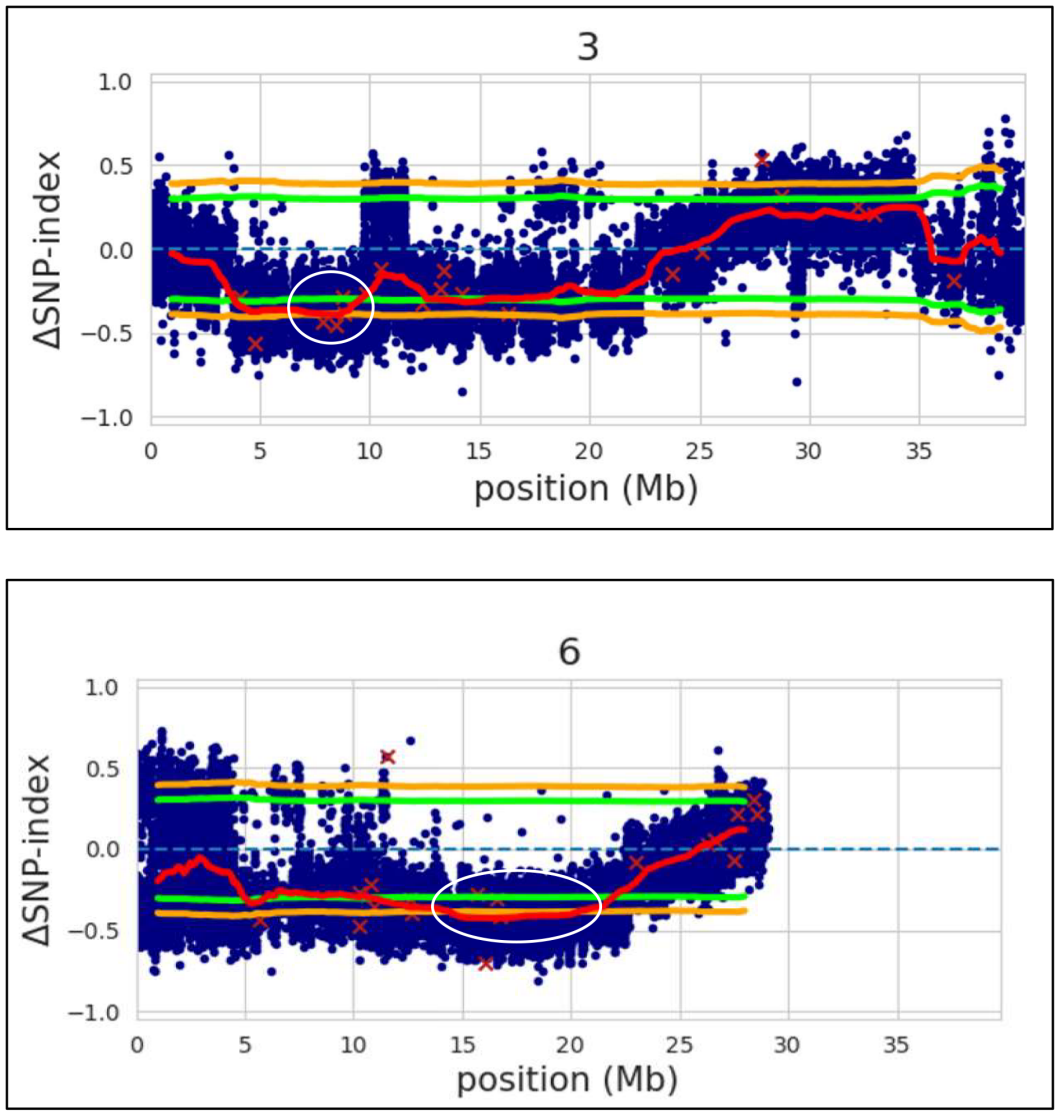
Δ(SNP-index) with statistical confidence intervals (orange, 99%; green, 95%). The major QTL identified on chromosome 6 and a minor QTL region on chromosome 3 of cucumber by QTL-seq (shown in white outline).

**Figure 5:**
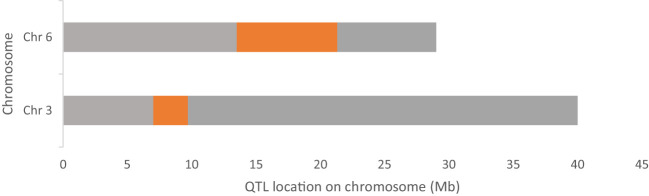
Chromosome 3 and 6 of cucumber showing the identified QTL region for parthenocarpy trait (in orange-coloured regions).

**Figure 6:**
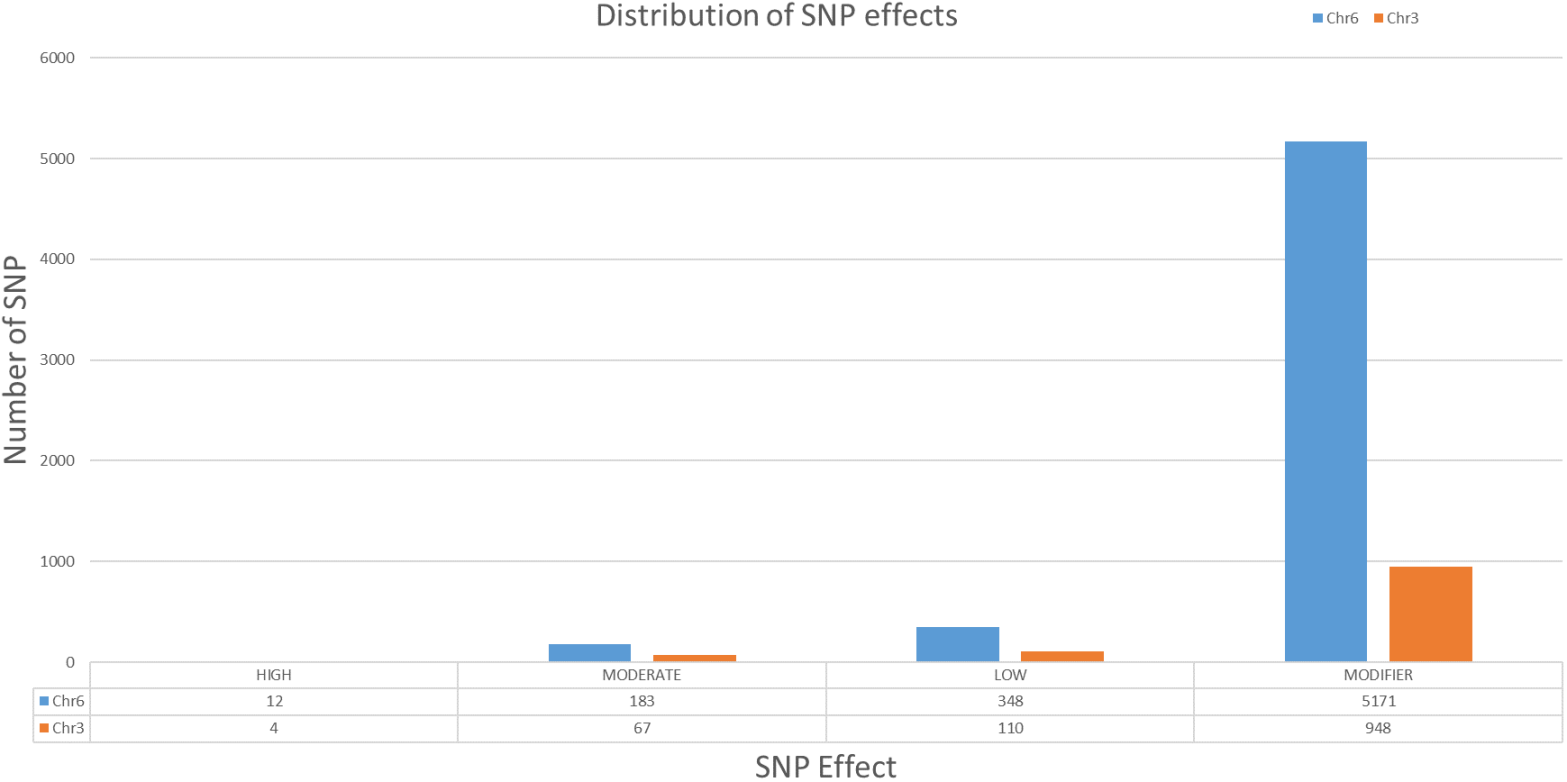
Distribution of SNP according to the effect in chromosome 3 and 6 of cucumber.

After alignment and filtering of clean reads followed by variant calling using BWA, SAM tools and BCF tools, two QTL regions related to parthenocarpy were detected. The major QTL was detected on chromosome 6 while a minor QTL region was detected on chromosome 3 (**Figure 4**; **Supplementary Table 1**). SNP index of the parthenocarpic and non-parthenocarpic bulks is presented in **Supplementary Figure 2**. For both the regions, 99% confidence interval was considered. The region covered under the QTL region of chromosome 6 expanded from 13,500,000 till 21,300,000 and 7,000,000 till 9,700,000 for chromosome 3 (**Figure 5**; **Table 3**). A total of 5714 variants (SNP and Indels) were identified in chromosome 6 while we found 1129 variants on chromosome 3 (**Supplementary Table 2**). To view the effect of these variations on the protein sequence, we used the SNPeff software using *Cucumis sativus* database. All the identified variations were divided into several categories based on the impact on the protein. These categories are High, Moderate, Low and Modifier. Chromosome 3 has 4, 67, 110 and 948 SNPs in each category, respectively while chromosome 6 has 12, 183, 348 and 5171 SNPs, respectively (**Figure 6**; **Supplementary Table 2**).

**Table 3:** Identified QTL regions in cucumber for parthenocarpy and their distribution in chromosome 3 and 6.

Chromosome	QTL	Start	End	Length (bp)	nSNPs
*Parth3.1*	1	7,000,000	9,700,000	2,700,000	1129
*Parth6.1*	2	13,500,000	21,300,000	7,800,000	5714

### Molecular mapping of parthenocarpy through conventional approaches

**Figure 7:**
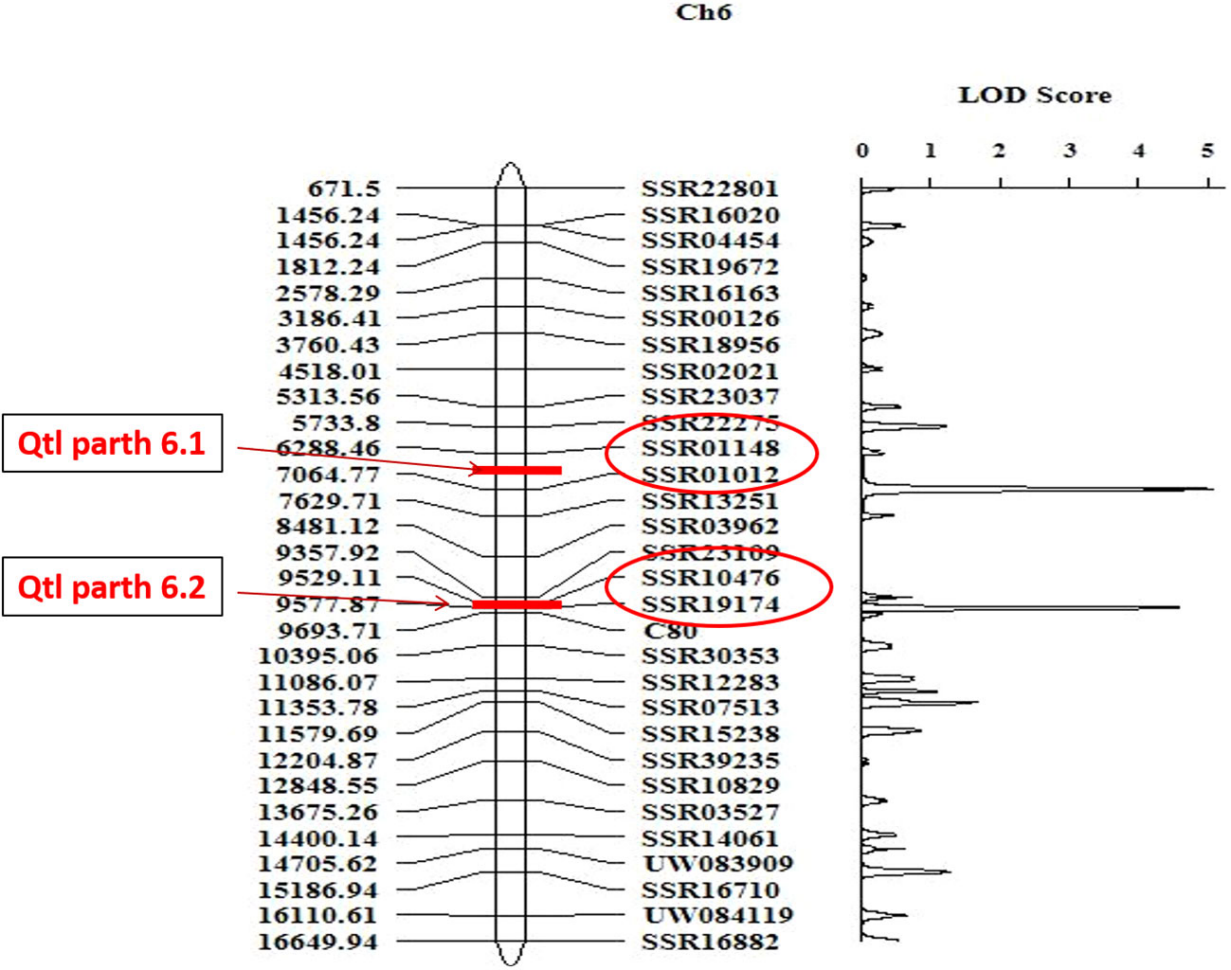
Linkage map of parthenocarpic locus on chromosome 6, constructed using SSR markers. Marker names, LOD score are depicted on the right side of the estimated map and the genetic distances shown in cM.

A total of 1285 SSR, Indel and CAPS markers were screened for parthenocarpy in polymorphic survey between PU and PPC-6 parental lines to identify polymorphic markers with ability to distinguish the parental lines. The markers were selected for all linkage groups of cucumber. A total of 1285 markers were used for parental polymorphic survey and among them 123 (11.28%) were polymorphic among the parental lines and produced clear and easily identifiable amplicons (**Supplementary Table 3**). The F_2_ mapping population comprising of 94 individuals, were genotyped using selected polymorphic markers and respective F_2:3_ progenies were evaluated for parthenocarpy. A total of 123 polymorphic markers were used for linkage analysis and others were rejected, due to non-amplification, missing data and difficulty in scoring. To confirm the parthenocarpic locus, specificity of the markers genotyping data of these 123 polymorphic markers were used for the construction of linkage map of parthenocarpic locus using Inclusive Composite Interval Mapping (ICIM) method of Ici Mapping (4.1.0.0) software at LOD threshold of 3.0 (Lander et al., 1987) (**Figure 7**). The linkage map of these polymorphic 123 SSRs, Indels, CAPS markers is presented in **Supplementary Figure 3**. Out of total 123 markers,10 markers were mapped on 1^st^ linkage group, 20 on 2^nd^ linkage group, 15 on 3^rd^ linkage group, 12 on 4^th^ linkage group, 17 on 5^th^ linkage group, 30 on 6^th^ linkage group and 19 on 7^th^ linkage group (**Supplementary Figure 4**). Two major effect QTLs associated with parthenocarpy (*Parth6.1* and *Parth6*.2) were mapped to chromosome 6 (**Figure 7**). These two QTLs had LOD scores of 5.06, 4.59 and phenotypic variance of 16.69% and 12.93%, respectively. The additive effects of *Parth6.1* and *Parth6.2* were -13.71 and -12.49, respectively indicating the contribution of the parthenocarpy trait from male parent PPC-6. The markers flanking *Parth6.1* locus were, SSR 01148 and SSR 01012, spanning a distance of 5.0 cM. The markers flanking *Parth6.2* locus were SSR10476 and SSR 19174, spanning a distance of 5.0 cM. The results from this experiment depicted that markers SSR 01148, SSR 01012, SSR10476, and SSR 19174 on chromosome 6 are closely associated with parthenocarpic traits in cucumber.

### Functional annotation of the identified QTL regions

**Figure 8:**
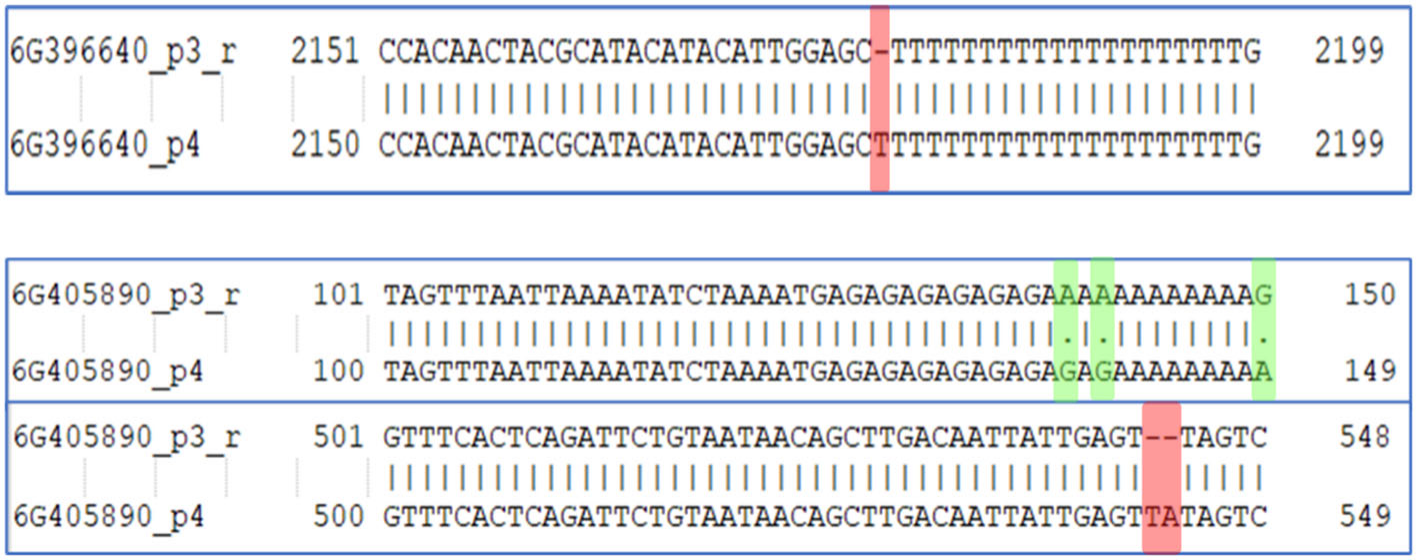
Variation in the *Csa_6G396640* and *Csa_6G405890* genes associated with indole-3-pyruvate monooxygenase YUCCA11 and Auxin response factor 16, respectively in the parents PU (p3_r) and PPC-6 (p4).

To identify the major genes, present in these regions, a reference genome annotation file of cucumber (http://ftp.ebi.ac.uk/ensemblgenomes/pub/release-53/plants/gff3/cucumis_sativus) was used. A total of 998 genes were present in chromosome 6, while 485 genes were present in chromosome 3 of the QTL region (**Supplementary Sheet 4**). Among the identified genes, majority of them were under the category of hypothetical protein. Two genes, *Csa_6G396640* and *Csa_6G405890* designated as probable indole-3-pyruvate monooxygenase YUCCA11 and Auxin response factor 16, respectively were the potential candidate genes associated with auxin biosynthesis in plants which is crucial in parthenocarpic fruit development in cucumber. *Csa_6G396640* gene showed only one variation (insertion) at position 2179 in PU while in case of *Csa_6G405890*, more variations were observed between the two parents which includes both SNPs and few INDELs (**Figure 8**).

## Discussion

Parthenocarpic fruit development in cucumber is extremely important for its cultivation under protected condition. Studies on inheritance of parthenocarpy by different workers depicted its complex genetics and number of genes/QTLs associated with resistance to parthenocarpy. In cucumber, parthenocarpy is facultative in nature and extent of parthenocarpy varies across different developmental stages of the plants (Joldersma and Liu, 2018). Pike and Peterson (1969) have reported that an incomplete dominant gene, *P* determines parthenocarpy in cucumber. They have postulated that development of early parthenocarpic fruits in the lower nodes is controlled by the dominant homozygous state, *PP* while late parthenocarpy and lower extent of parthenocarpy are represented by the heterozygous state, *Pp*. Whereas, homozygous recessive state, *pp* is responsible for non-parthenocarpic fruit development. In the homozygous condition, *PP* produces parthenocarpic fruits early, with the first developing generally by the fifth node. Heterozygous *Pp* plants produce parthenocarpic fruits later than homozygous plants and are fewer in number. The homozygous recessive *pp* produces no parthenocarpic fruits. Besides, several other studies have also reported monogenic control of parthenocarpy in cucumber (Hawthorn and Wellington, 1930; Kvasnikov et al., 1970; Juldasheva, 1973; Meshcherov and Juldasheva, 1974). Later on, most of the studies reported polygenic control of parthenocarpy (El-Shawaf and Baker, 1981; Sun et al., 2006; Yan et al., 2009; Lietzow et al., 2016; Wu et al., 2016). In the present study, we have recorded that there was varied types of parthenocarpy in the F_2_ progenies although the parthenocarpic parent, PPC-6 was early parthenocarpic type and started fruiting from 3-5 nodes onwards. We have recorded the plants as early parthenocarpic when fruiting started from 5^th^ node onwards and late parthenocarpy when parthenocarpic fruit set was recorded after 10^th^ node and considered all the early and late-type plants as parthenocarpic. Based on these observations, the parthenocarpy was found to be controlled by single recessive gene. The present information supported the earlier observation by Pike and Peterson (1969) which postulated that homozygous dominant, heterozygous and homozygous recessive forms are responsible early parthenocarpy, late parthenocarpy and non-parthenocarpy, respectively. However, it was evident there was a significant contribution of the back-ground evidenced from the extent of parthenocarpy and therefore, it is possible to introgress parthenocarpy traits in different elite and non-conventional genotypes of cucumber through marker-assisted back-cross breeding with identification and development of molecular markers closely associated with parthenocarpic trait.

In the recent times, discovery of molecular markers and physical map construction is greatly facilitated by advancement in next-generation sequencing technology (Cao et al., 2021). QTL-seq combines the next-generation sequencing technology with BSA for rapid detection of QTLs for any particular trait and facilitate development of closely associated molecular markers and identification of candidate genes. Thereafter, QTL-seq has been widely used for the detection of QTLs, identification of closely linked molecular markers and identification of candidate genes for number of traits in different crops (Singh et al., 2016; Wang et al., 2016; Wei et al., 2016; Chen et al., 2017; Wen et al., 2019; Arikit et al., 2019; Li et al., 2020). In cucumber, QTL-seq has been used successfully for the identification of QTL for early flowering traits (Lu et al., 2014), flesh thickness (Xu et al., 2015), sub-gynoecy sex expression (Win et al., 2019), pre-harvest sprouting of the seeds (Cao et al., 2021) and resistance to powdery mildew (Zhang et al., 2021). Based on the QTL-seq results, two major QTLs, one each in chromosomes 3 and 6 were identified based on the Δ(SNP-index). The QTL, *Parth3.1* was *Parth6.1* were spanned 2.7 Mb on chromosome 3 and 7.8 Mb on chromosome 6, respectively. Besides, a large number of variants were detected in both the genomic regions in the form of SNPs and InDels. The identified SNPs and InDels in the genomic regions detected through QTL-seq will be extremely useful in the development of molecular markers and fine mapping of the genomic region associated with parthenocarpy in cucumbers. However, the QTL region identified through QTL-Seq are often not very precise and needs further validation and authentication through additional method of molecular mapping (Xu et al., 2017). Therefore, we have employed mapping of parthenocarpy through conventional F_2:3_ population. Based on the QTL-seq results, two major QTLs, one each in chromosomes 3 and 6 were identified.

In cucumber, systematic efforts have been made for molecular mapping of number of qualitative traits but for quantitative traits like parthenocarpy progress is slow and hence very few public sector parthenocarpic varieties/hybrids are available in market. Now-a-days, role of marker-assisted selection (MAS) is increasing in conventional plant breeding (Miao et al., 2011). Due to narrow genetic base and low polymorphism, around 30 linkage maps have been constructed (Kennard et al., 1994). These linkage maps involved use of RAPDs (Randomly Amplified Polymorphic DNA) or AFLP (Amplified Fragment Length Polymorphism) (Fukino et al., 2008 and Yuan et al., 2008) that are not breeder friendly. Hence, co-dominant markers like SSR/InDel are best suited for marker-assisted breeding and are breeder friendly.

Two major QTLs for parthenocarpy at chromosome 6 (*Parth6.1* & *Parth 6.2*) were identified using the F_2:3_ mapping population. Locus *Parth6.1* was flanked by SSR01148 and SSR01012 with a LOD score of 5.06 and 16.69% of PVE (Phenotypic Variance Explained) reflecting that this locus is a major effect QTL. Second QTL *Parth 6.2* was flanked by SSR10476 and SSR19174 primers and LOD value was 4.59 with 12.93% of PVE explaining another major effect QTL. Previously, Lietzow et al. (2016) identified seven QTLs associated with parthenocarpic fruit set, one on each chromosomes 5 and 7 (*parth5.1* and *parth7.1*) and two on chromosome 6 (*parth 6.1* and *parth 6.2*) were consistently identified in all experiments. Wu et al. (2016) identified seven novel QTLs on chromosomes 1, 2, 3, 5 and 7. The identification of QTLs is a valuable resource for cucumber breeders for the development of parthenocarpic cultivars (Dey et al., 2022). Molecular markers flanking major effect parthenocarpy QTLs can prove useful in the Marker Assisted Breeding (MAB) programme. However, there is need to further saturate linkage map to narrow-down genetic distance between flanking molecular markers to get markers better suited for foreground selection in endeavour of higher/quality production of cucumber.

Parthenocarpy is a complex trait and determined by interaction of large number of metabolic pathways interlinked with each other. Among the different metabolism, auxin, gibberellins and cytokinins are reported to play key role determining parthenocarpic fruit set in cucumber (Li et al., 2014; Su et al., 2021; Sharif et al., 2022; Gou et al., 2022). Cross-talk between the important phytohomones in determining parthenocarpy in PPC-6 was recently reported by Mandal et al. (2022). The QTL region identified through QTL-seq had two important genes with a possible association with parthenocarpic phenomenon. Indole-3-pyruvate monooxygenase YUCCA11 (*Csa_6G396640*) was found to be have one SNP in the non-parthenocarpic parent, PU when compared with the parthenocarpic reference genotype. Besides, the auxin response factor 16 (*Csa_6G405890*) present in the QTL region also showed variation in terms of Indels and SNPs in the parental lines. These two identified genes with key role in auxin biosynthesis could be possible candidate genes for induction of parthenocarpy in cucumber. Auxin, through its influence in cell division and expansion is key determinant in development of fleshy fruits and reported to be integral part in the initial signal for fertilisation and increased fruit (Godoy et al., 2021). After parthenocarpic fruit set their further development is influenced by auxins which was evidenced by the upregulation of the auxin biosynthesis-related genes in the later stages of fruit development in parthenocarpic genotypes in our earlier study (Mandal et al., 2022). Indole-3-pyruvate is one of the important routes for tryptophan-dependent auxin biosynthesis which is believed to be common in all plants (De Smet et al., 2011). Auxin is the key phytohormone besides gibberellins and cytokinin reported to play important role in the induction of parthenocarpy (Sharif et al., 2022). Among the different auxin biosynthesis pathways, the role of *Trp-IPyA* (tryptophan-indole-3-pyruvic acid) in parthenocarpic fruit development has been reported by several workers. The role of the *YUCCA10*, *PavYUCCA10*, *SlTAR1, ToFZY2, ToFZY3* and *PARENTAL ADVICE-1 (PAD-1)* genes in parthenocarpic fruit development of loquat, tomato and eggplants have been reviewed in details by Sharif et al. (2022). However, narrow down of the QTL region through fine mapping is required for the precise identification of candidate genes associated with parthenocarpy in cucumber.

## Conclusion

In cucumber parthenocarpic fruit set is extremely important trait facilitated large scale protected cultivation worldwide. In one commercially cultivated parthenocarpic genotype, PPC-6, it was found that, single incomplete dominant gene control this trait in spite of significant effect of genetic back-ground in expression of parthenocarpy. QTL-seq analysis in combination with conventional mapping using F_2:3_ population identified one major effect QTLs, *Parth6.1*. The flanking markers, SSR01148 and SSR 01012 for Parth6.1 locus were identified for their use in marker-assisted back-crossing programme. Two major genes, *Csa_6G396640* and *Csa_6G405890* designated as probable indole-3-pyruvate monooxygenase YUCCA11 and Auxin response factor 16, respectively associated with auxin biosynthesis as potential candidate genes. The study provides insight about the genetics and genomic regions, closely associated markers and possible candidate genes associated with parthenocarpy in PPC-6 for functional genomics studies and future fine mapping.

## Data availability statement

The data presented in the study are deposited in the NCBI repository, accession number PRJNA885599, https://www.ncbi.nlm.nih.gov/bioproject/PRJNA885599.

## Author contributions

Conceived theme of the study and designed experiment: ShyD. Data curation: PS, ShyD, MI, SJ, GS. Investigation: ShiD, ShyD, KK, BG. Resources: ShyD, TB, AM. Supervision: ShyD, AM, TB, GS. Visualization: ShyD, TB, AM. Writing original draft: ShiD, ShyD, SJ. Review and editing: ShyD, TB, GS, MI. All authors contributed to the article and approved the submitted version.

## Supplementary material

Supplementary Figure 1 Fruit setting pattern in the F_1_ hybrid involving Pusa Uday × Pusa Parthenocarpic Cucumber-6.

Supplementary Figure 2 SNP indices of **(A)** Parthenocarpic bulk (PB) and **(B)** Non-partyhenocarpic bulk (NPB) for each chromosomes.

Supplementary Figure 3 Linkage map of parth locus on chromosome 6, constructed using SSR markers. Marker names, LOD score are depicted on the right side of the estimated map and the genetic distances shown in cM on the left are calculated using software IciMapping ver. 4.1.0.

Supplementary Figure 4 Distribution of primers across the seven linkage groups and frequency of the amplified and polymorphic markers used for the study.

Supplementary Figure 5 Amplification pattern of the selected markers closely associated with parthenocarpy in cucumber genotype, PPC-6.

Supplementary File 1 Identified QTL regions in chromosome 3 and 6 through QTL-seq.

Supplementary File 2 Identified SNPs and InDels in the QTL regions of chromosome 3 and 6, their impact and associated genes.

Supplementary Table 3 List of the polymorphic markers used for construction of linkage map and molecular mapping of parthenocarpy using F2:3 population.

Supplementary File 4 Functional annotation of the genes present in the QTL regions in chromosome 3 and 6.
