# Supplementary material for: Identification of a major QTL, Parth6.1 associated with parthenocarpic fruit development in slicing cucumber genotype, Pusa Parthenocarpic Cucumber-6
Source: Front Plant Sci. 2022 Dec 14;13:1064556. doi: 10.3389/fpls.2022.1064556 (PMC9795203; doi:10.3389/fpls.2022.1064556)
Supplement: Supplementary Figure 1 — Fruit setting pattern in the F1 hybrid involving Pusa Uday × Pusa Parthenocarpic Cucumber-6. [file DataSheet_1.zip › Image 5.PPTX]

## Slide 1
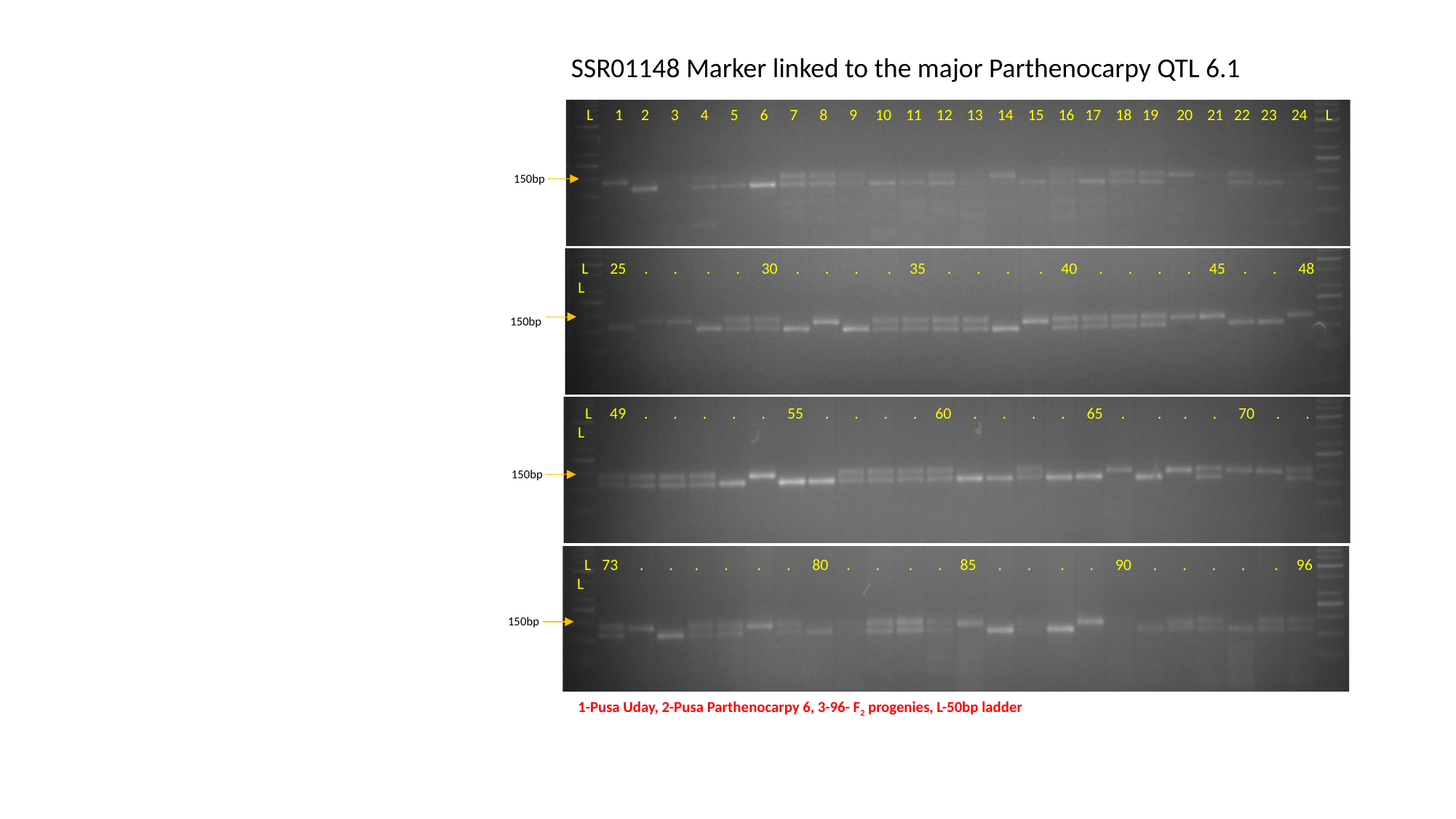

SSR01148 Marker linked to the major Parthenocarpy QTL 6.1
 L 1 2 3 4 5 6 7 8 9 10 11 12 13 14 15 16 17 18 19 20 21 22 23 24 L
150bp
 L 25 . . . . 30 . . . . 35 . . . . 40 . . . . 45 . . 48 L
150bp
 L 49 . . . . . 55 . . . . 60 . . . . 65 . . . . 70 . . L
150bp
 L 73 . . . . . . 80 . . . . 85 . . . . 90 . . . . . 96 L
150bp
1-Pusa Uday, 2-Pusa Parthenocarpy 6, 3-96- F2 progenies, L-50bp ladder

## Slide 2
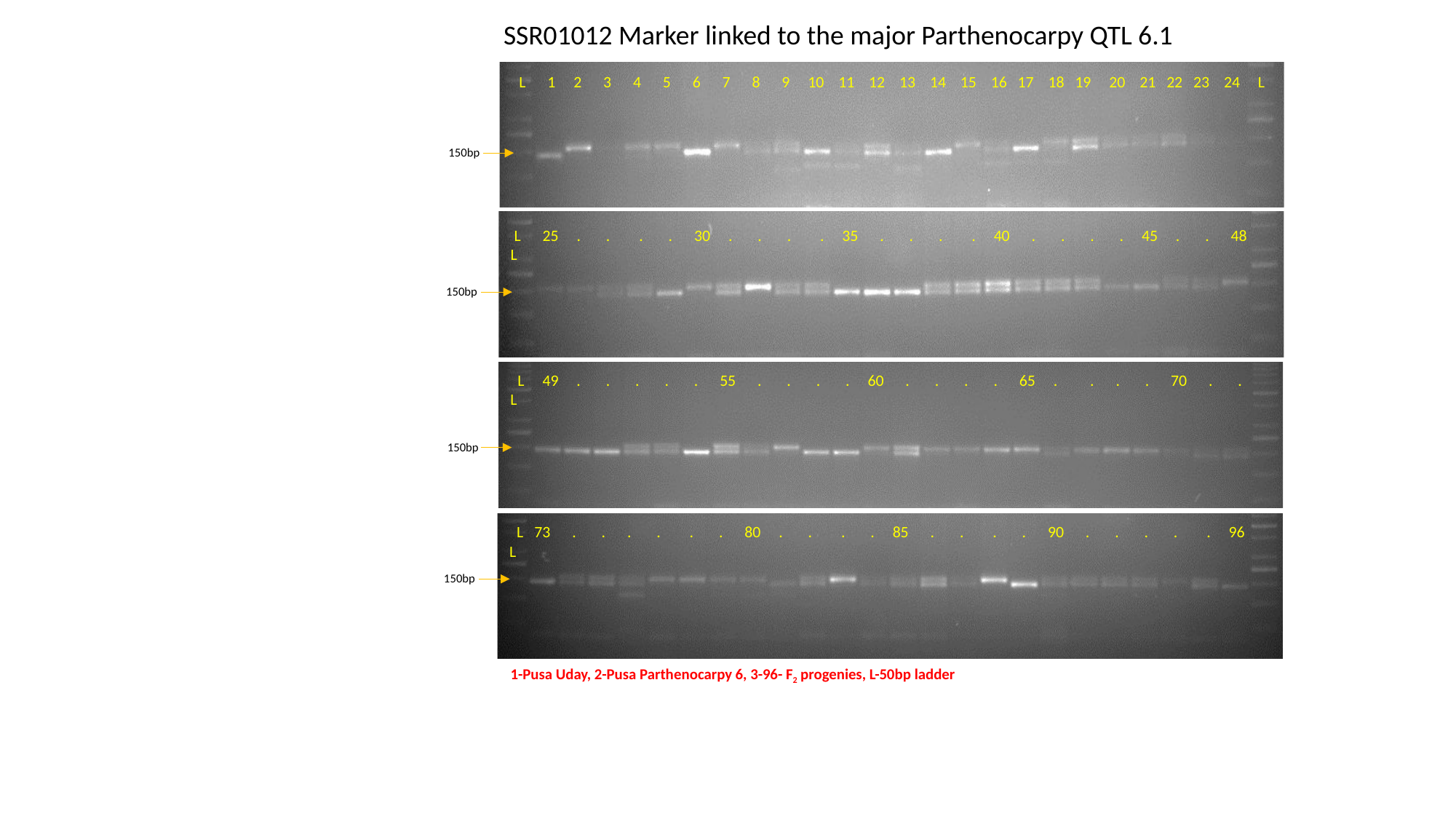

SSR01012 Marker linked to the major Parthenocarpy QTL 6.1
 L 1 2 3 4 5 6 7 8 9 10 11 12 13 14 15 16 17 18 19 20 21 22 23 24 L
150bp
 L 25 . . . . 30 . . . . 35 . . . . 40 . . . . 45 . . 48 L
150bp
 L 49 . . . . . 55 . . . . 60 . . . . 65 . . . . 70 . . L
150bp
 L 73 . . . . . . 80 . . . . 85 . . . . 90 . . . . . 96 L
150bp
1-Pusa Uday, 2-Pusa Parthenocarpy 6, 3-96- F2 progenies, L-50bp ladder

## Slide 3
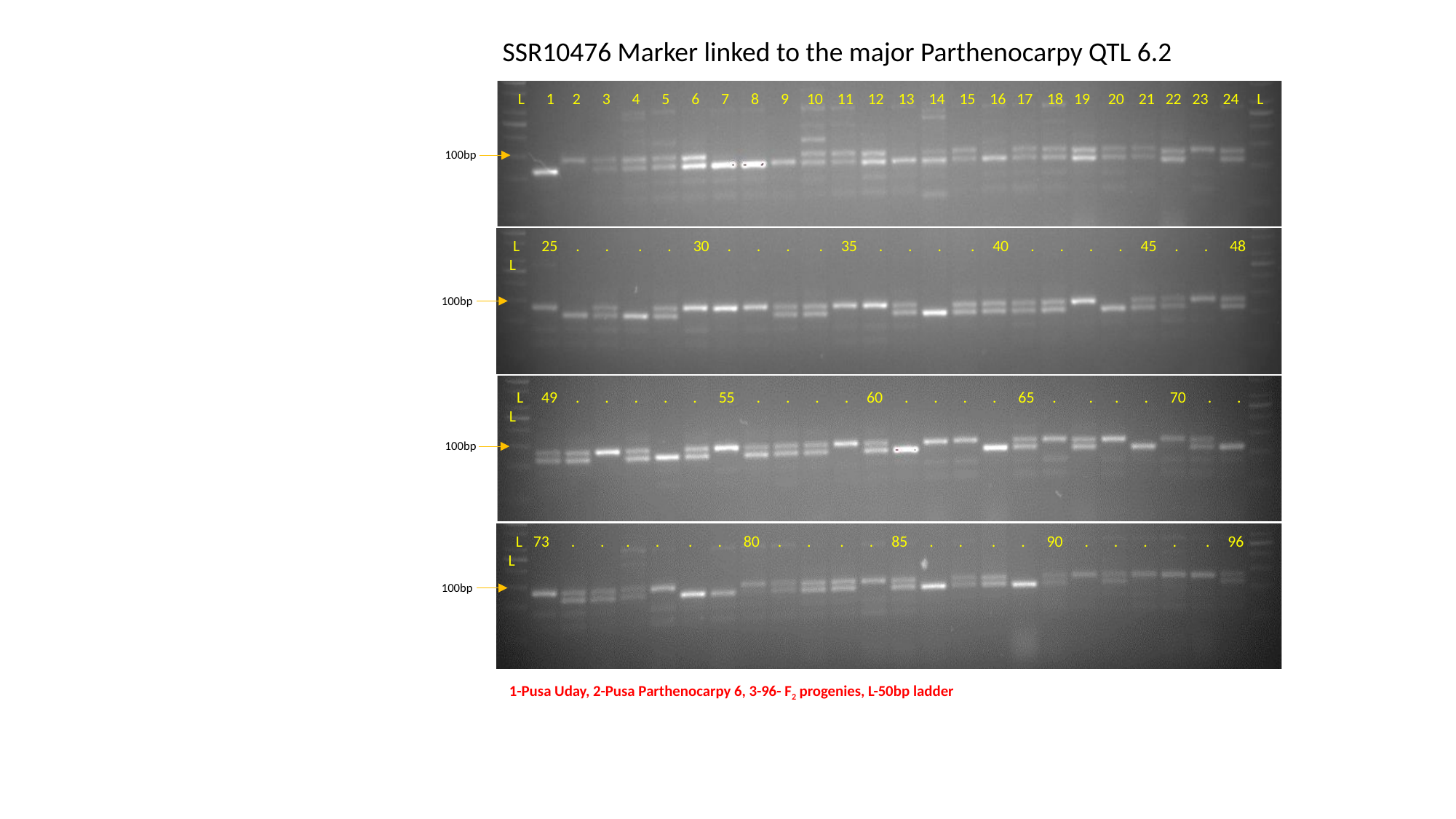

SSR10476 Marker linked to the major Parthenocarpy QTL 6.2
 L 1 2 3 4 5 6 7 8 9 10 11 12 13 14 15 16 17 18 19 20 21 22 23 24 L
100bp
 L 25 . . . . 30 . . . . 35 . . . . 40 . . . . 45 . . 48 L
100bp
 L 49 . . . . . 55 . . . . 60 . . . . 65 . . . . 70 . . L
100bp
 L 73 . . . . . . 80 . . . . 85 . . . . 90 . . . . . 96 L
100bp
1-Pusa Uday, 2-Pusa Parthenocarpy 6, 3-96- F2 progenies, L-50bp ladder
