# Supplementary figures and images for: Identification of a major QTL, Parth6.1 associated with parthenocarpic fruit development in slicing cucumber genotype, Pusa Parthenocarpic Cucumber-6
Source: Front Plant Sci. 2022 Dec 14;13:1064556. doi: 10.3389/fpls.2022.1064556 (PMC9795203; doi:10.3389/fpls.2022.1064556)

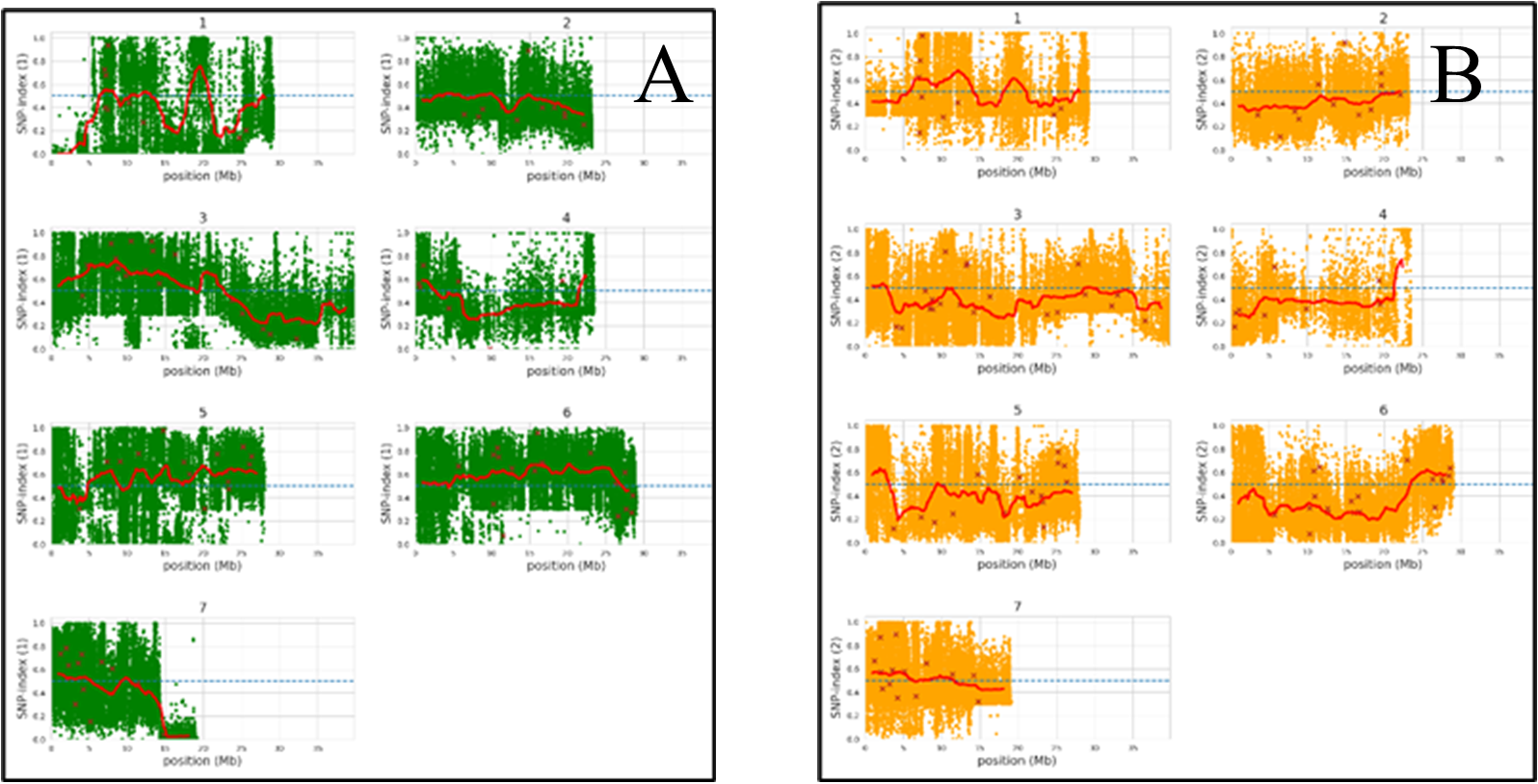

Supplement: Supplementary Figure 1 — Fruit setting pattern in the F1 hybrid involving Pusa Uday × Pusa Parthenocarpic Cucumber-6. [file DataSheet_1.zip › Image 1.TIF]

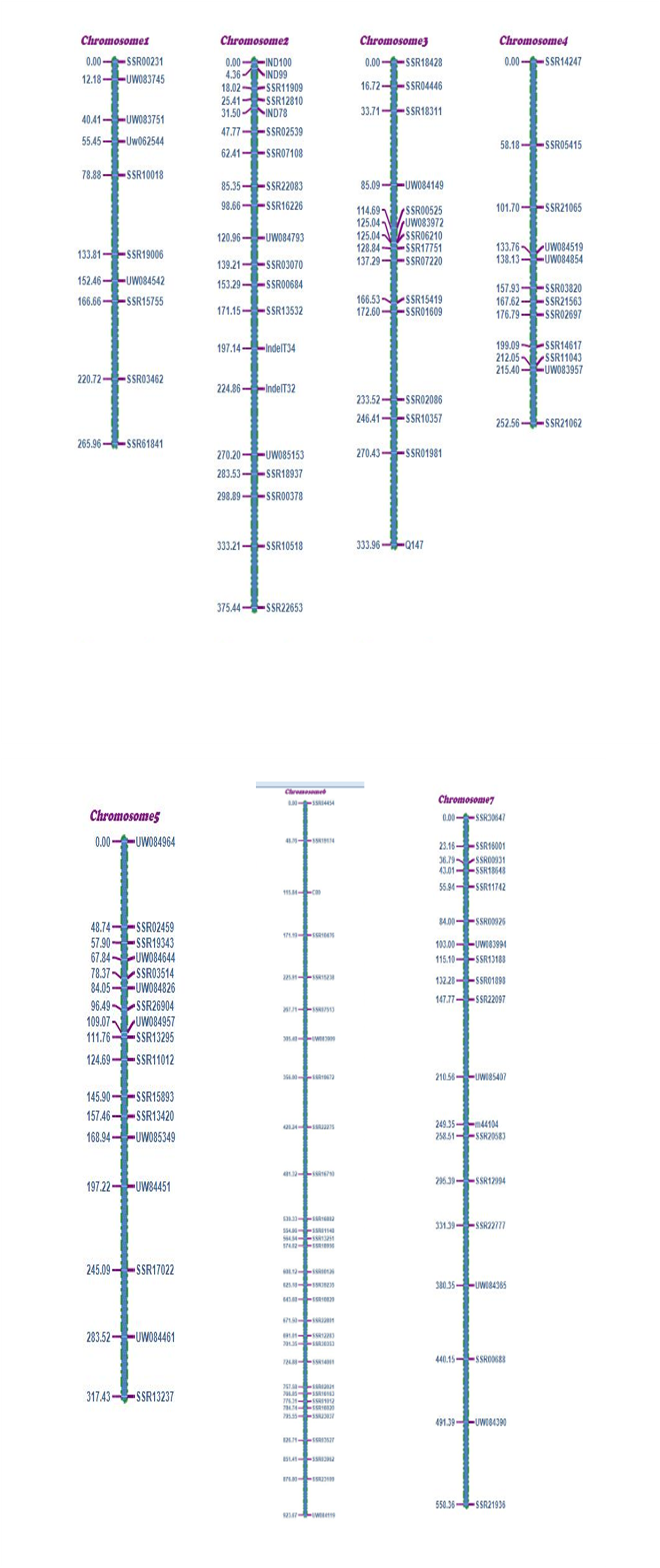

Supplement: Supplementary Figure 1 — Fruit setting pattern in the F1 hybrid involving Pusa Uday × Pusa Parthenocarpic Cucumber-6. [file DataSheet_1.zip › Image 2.TIF]

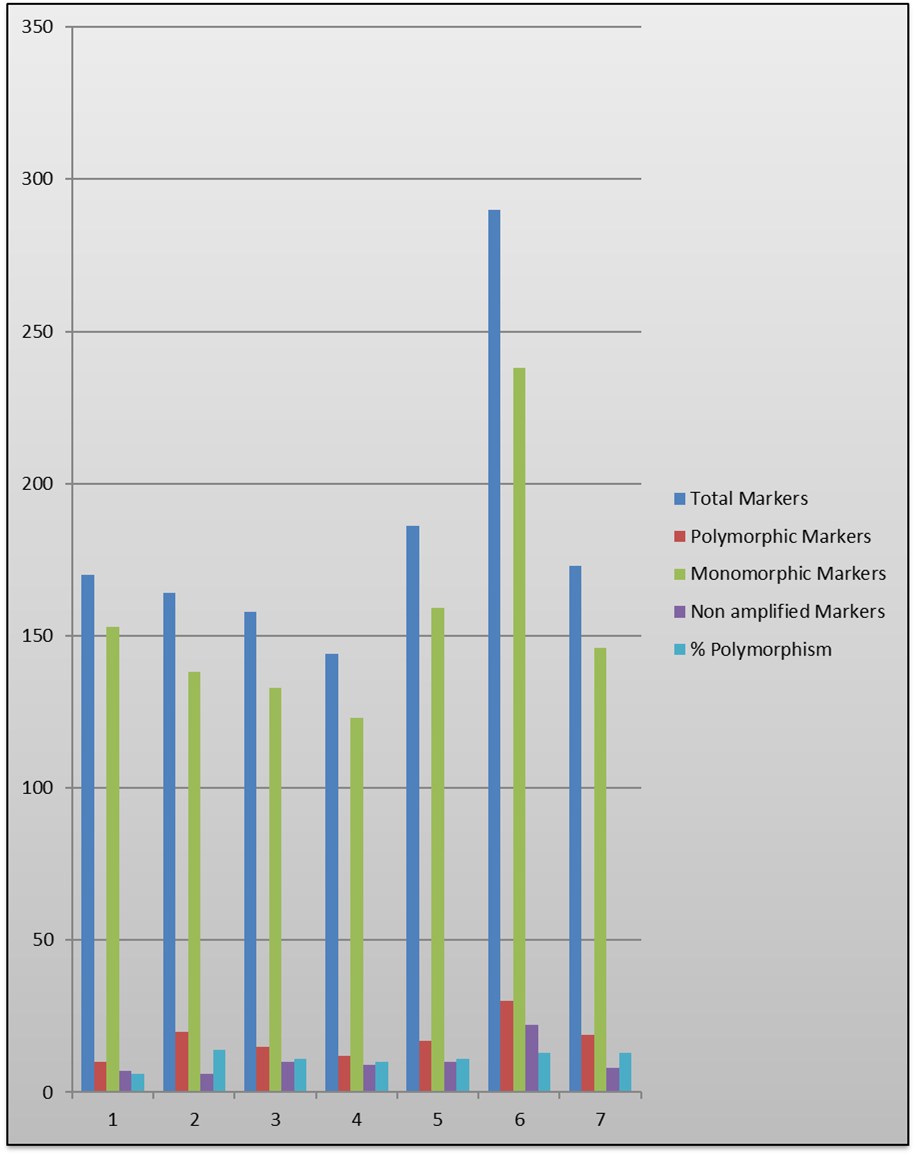

Supplement: Supplementary Figure 1 — Fruit setting pattern in the F1 hybrid involving Pusa Uday × Pusa Parthenocarpic Cucumber-6. [file DataSheet_1.zip › Image 3.TIF]

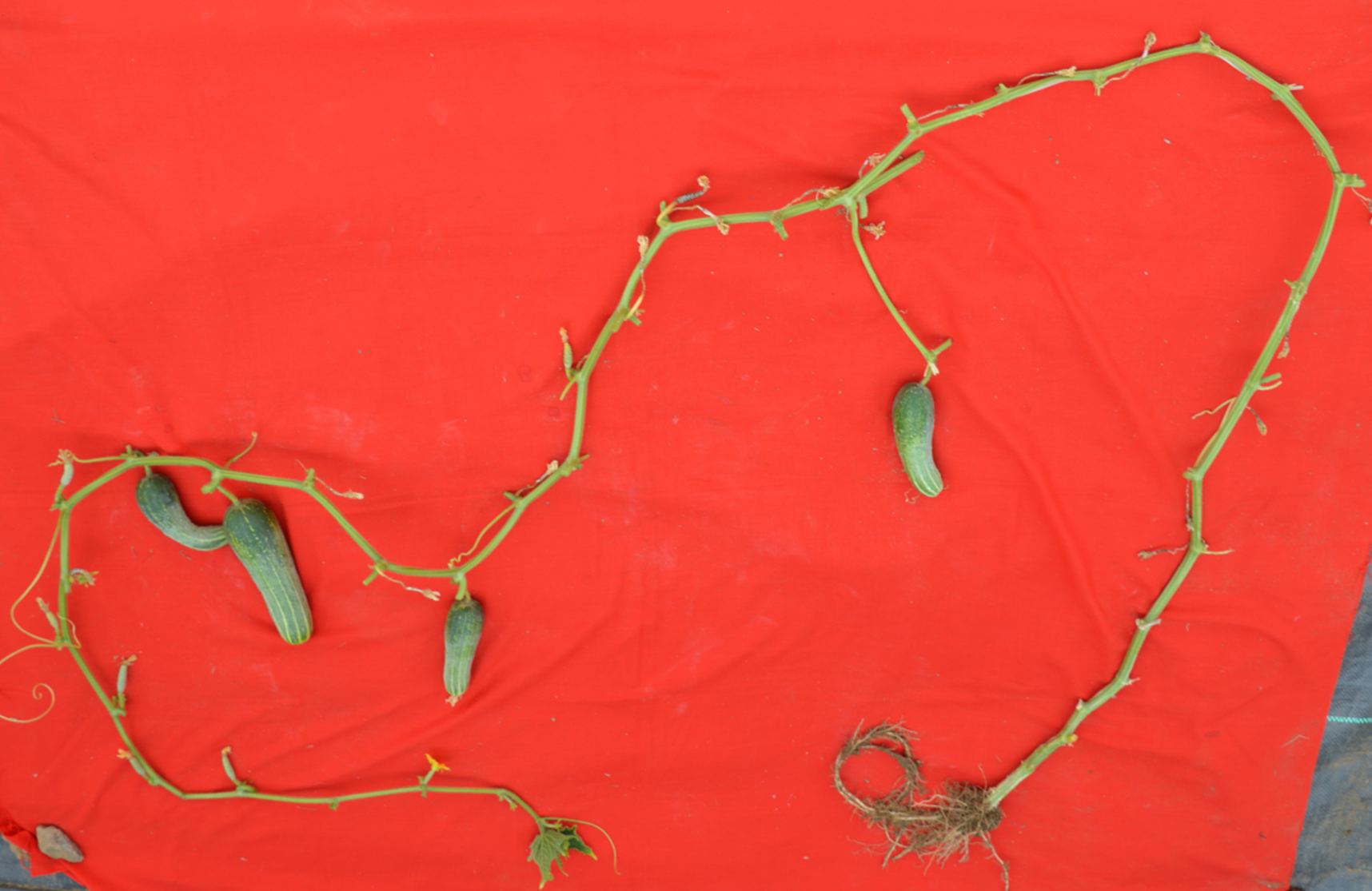

Supplement: Supplementary Figure 1 — Fruit setting pattern in the F1 hybrid involving Pusa Uday × Pusa Parthenocarpic Cucumber-6. [file DataSheet_1.zip › Image 4.TIF]
