# Supplementary material for: Identification of a major QTL, Parth6.1 associated with parthenocarpic fruit development in slicing cucumber genotype, Pusa Parthenocarpic Cucumber-6
Source: Front Plant Sci. 2022 Dec 14;13:1064556. doi: 10.3389/fpls.2022.1064556 (PMC9795203; doi:10.3389/fpls.2022.1064556)
Supplement: Supplementary File 1 — Identified QTL regions in chromosome 3 and 6 through QTL-seq. [file DataSheet_2.zip › Table 3.DOCX]

**Supplementary Table 4.** Forward and reverse primer sequence of polymorphic markers used for molecular mapping of cross PU X PPC-6

| **Chr No.** | **Primer name** | **Forward Sequence** | **Reverse Sequence** |
| --- | --- | --- | --- |
| 1 | SSR00231 | GAGGTTGGGAAATTGGGAAT | TATTCAAACACAAAGCCCGC |
| 1 | UW083745 | TCTTTTGATCATTCAATACACTACCA | TGTTTTACACAAAGGTTATGATTAGGA |
| 1 | UW083751 | GTGAAGATACGTTGTTGTCAAAAT | CCGTTGTATCCATTTCAAGATGT |
| 1 | UW062544 | GCTACCGACACCTTTGCTTT | AGCAACCCACCAAGAAGTTG |
| 1 | SSR10018 | CTTTTGTTCTTGTGGAATGTGA | ATTTGGGGATGGAGAGGTTC |
| 1 | SSR19006 | GAAATTATGATGAATACCACAAACCA | TTCACATGCCAAACTGATCTTT |
| 1 | UW084542 | CTGCTTCTTCCCATCCAATG | TCAATCCAAACAGACATTTTCG |
| 1 | SSR15755 | GTGCCTCAGTCGGTTTTGTT | GGGAGGGGAAGGATTCAATA |
| 1 | SSR03462 | CACGCGAGAGGAGAGGATAG | GAAACAAAATCTGGGGTCCA |
| 1 | SSR16841 | TTTGATGACAACAATCTTCATCG | CCTGTCGACAACATGGATTCT |
| 2 | IND100 | TTGAGTTTGATAGCCGGATAGAGC | ACGTGGCAAGCTCTTTTTAATGAC |
| 2 | IND99 | TTATTTATCTCCTTGGCTGGGTTG | GGCTATTAGCATATAGCAACAAGGTAGG |
| 2 | SSR11909 | AATAATACCAGTGGCCCCATC | AAAGCTCCCTCCTCCCCTAC |
| 2 | SSR12810 | TTCCCACAAAACAAATCTTGG | TTTTGGAGAGAAAAGGTTGGA |
| 2 | IND78 | CCTCAAGAAGATTGGCAATGAAGT | CCTGTTTTTGGGCTTTCTAAGGTT |
| 2 | SSR02539 | AAAAATGATCAGCTCGATGAAA | GCAAGCGCTTTCCAATCTAT |
| 2 | SSR07108 | TAAGCAATTCCAGGAGAGGG | GTTCTTTGATGGGTGCCTGT |
| 2 | SSR22083 | TTATGCAACCATTGGAGAAGG | TGATTTGTTGTCTGTGCCAA |
| 2 | SSR16226 | TTAAAATTCCCAACGGAAACC | TGATGGGAGAAAGGTAAACAAGA |
| 2 | UW084793 | CAAAGCATTGTGTGATGGTC | TTACTTCCCATGGTCCAGGT |
| 2 | SSR03070 | GCTAACACTACCCGCTGCTT | AACAGAAAGAGAATCGGGGG |
| 2 | SSR00684 | AAGGCCAAAAGACTATGCGA | CATCCCTTGCATCTCCACTT |
| 2 | SSR13532 | AAACCCAAGAATTGTAAACCCA | TGATCCATTTCTATTCCTAACATTGA |
| 2 | Indel T34 | GAACTCCAAGCTCTGTCACCATTT | TGGTTGATCATAGATGTTTATCCATTG |
| 2 | IndelT32 | CGGCGAAAGAGACTGAAAAGTAAA | CTCACTATTCCAATTCCCAACCG |
| 2 | UW085153 | TTTATATTTAGGGAAACACACACG | CAAAAATCATGTGTAACTAAGCAAA |
| 2 | SSR18937 | TTACTCCAAAGATGCTGGGC | CATTTGACCGAATCTTGACTTT |
| 2 | SSR00378 | TCCCTAAAATTTCGACAACCC | TTAGTATGGCTTGAACACCCA |
| 2 | SSR10518 | TCTAATTCGCTCCGGATGAT | TTGCAGCGAACAATCCTGTA |
| 2 | SSR22653 | TGAATTTCTTTGGTGGATTCAA | ACCTCCCATGGCAATCTACA |
| 3 | SSR18428 | CCATTCACTTCCTTTCCAGC | TGGTTTCAAGACCACCCTCT |
| 3 | SSR04446 | CAGTGTGCCAATACCACCAA | AAGTGACAATGGCAGGTCAA |
| 3 | SSR18311 | GCGGATCAGAGAGGAAACAG | GAAACAAACGTCCTCCTCCA |
| 3 | UW084149 | TCATCTGCTTCATTTCTTGGAA | GCTCCAGAATCCAAGATCCA |
| 3 | SSR00525 | GGGCAGTGAAATTGGAGGTA | TTAAATAATGGGCTGGGCTG |
| 3 | UW083972 | TCGAGGTGTATGGTTGTTGG | TTTTTGTGTTGTCTCACAGTTCA |
| 3 | SSR06210 | TTGGAAAAGTCGCCAAACTT | TCCATGTCTGCTTTTGATTCC |
| 3 | SSR17751 | TCCAACGGAAAATTACAAGGA | TCAATTGTTGGATTCATGTCAA |
| 3 | SSR07220 | CCATTGTCTCTTCCTTCGAAAA | CATCTGTTGTGGAGGCATTG |
| 3 | SSR15419 | ATGGCAAAGCCAAAAAGATG | TGTTGCAAATATTGCACCTTC |
| 3 | SSR01609 | TGGATGCATTCCAAACTTCA | TGCAAGCAGTGTTCGATGTT |
| 3 | SSR02086 | AACGACAGCGTTTCCTCACT | GGTATAATTGGGGCGATCCT |
| 3 | SSR10357 | AAGAAGTATGCGAGGTAAGGGA | GCCAAGAAGTAAGAAGTGGTTGA |
| 3 | SSR01981 | GTTTGGACGACTGTGTGGTG | GGGAGCATCTCCTCTTAGATATG |
| 4 | SSR14247 | TCACATCCATTTCGGACAAA | TCACCAGTGACCTGTGAAGAA |
| 4 | SSR05415 | GGGCATCATGACTAAATTCTCC | GTCTTCCTGGGTTAGTGGGG |
| 4 | SSR21065 | GGCTCCATATGCCAAATGAC | ATGGGTCCAGAGCTTTTTGA |
| 4 | UW084519 | GGTAAGAGATGATCTTCGAAAGG | TTCCATTCATATTCTTCCAATGC |
| 4 | UW084854 | AAGTTGGATTTAGGGGATGC | GGACCCCTACCTAATGCTCA |
| 4 | SSR03820 | AGAGGGCAAATTGGTGAATG | TCCATCCTGTATGATTTGAGTTG |
| 4 | SSR21563 | GCAAGGAGTGAACAAACGGT | GTTCTCCACCATAACCCCCT |
| 4 | SSR02697 | TGCTAACCCAACCAAACAAA | CTGCCATTTCAAGCTATGGG |
| 4 | SSR14617 | CAACTCCGGTTCAAAAGTTCA | TGTCTTCAATGCCCTTTCAG |
| 4 | SSR11043 | AGGTACGAAACAACGGCAAT | TCGCACTCACTCTTTACCGA |
| 4 | UW083957 | CCCTCTCAACCTCAAGATCG | AGGTACGAAACAACGGCAAT |
| 4 | SSR21062 | TCACACAACAACTCCATAACACA | GGCCGCGTAGTAGAGTGAAG |
| 5 | SSR15893 | ATGGGAGCTACACGTTGAGG | ATAGGCTTCCATTCCAGGGT |
| 5 | SSR13237 | AGGGAGTTGGAAGAGGTGGT | AGTGAAAACAGTCAGGAGGTGA |
| 5 | SSR03514 | TAGGGTCCCCTTCCCTCATA | GGGTACCCAAAAGCAAGTGA |
| 5 | SSR02459 | TCGGAAGATGGGTTATTTGG | TGACCCCTCACATTCTCTCC |
| 5 | SSR26904 | CCACCATGTTGTGCTTATCAA | GACCCTTCCAAAAGTAATAAACAA |
| 5 | SSR17022 | CTCAACAAGTTCGGTCCCAT | AATCAAATGTGGGTGGTGGT |
| 5 | UW084461 | GGCTACAGGGACATAAATACACTT | CGTTGTAATTACTTGGCCATCA |
| 5 | UW084826 | CTCCCTCTTAGCCCTCACCT | GACGAAAGTAAGAGAAAATTTAAGCA |
| 5 | UW084451 | GCTTCTTGTTCTTGGGAGGA | ACCCACTACCCACAACGAAA |
| 5 | SSR13420 | GGGTTTGGGTTGGTTTAGGT | CTTTCATCACACCCTCCTCC |
| 5 | UW085349 | AACAGTTCACGGAAGATCCAG | AACAAACACCGCTCTCAGTG |
| 5 | SSR13295 | AAATCCCCTCACCTCATTCC | TCAATTAAAGTAGACGTTTCGAATAA |
| 5 | UW084957 | CACAAACAAAGTTGCCAACAA | TCAATTGGGCACAAATCCTT |
| 5 | UW084964 | CATCATCTTCAAGCAATCGTACA | TGTTGGATTTGAGCACCAGA |
| 5 | SSR19343 | ACCACGTGTATCTTCGCCTC | TCAAATGCATTGAAGGCTGT |
| 5 | UW084644 | ATGGTCCCTTCTTTCTCCTTC | AACATCCTAGCCAATTCCTC |
| 6 | SSR22801 | GGGTGAGACATAGTTCTGTGTGAA | CTTGACCAAGAGGTCAAAGC |
| 6 | SSR16020 | ACACCATTTTTCATCGAGATTT | GGGATGAGGAGCAAATGGTA |
| 6 | SSR04454 | GGAAAGTGTTGTGTTGCTCTTG | GATCCATTTGATGCATTGCT |
| 6 | SSR19672 | AAGGCAGCAGAAAACTTGGA | CCCTCACTCTCGCTCACTCT |
| 6 | SSR16163 | CCAATATTTGCATATGGTTTATCA | CCAATATTTGCATATGGTTTATCA |
| 6 | SSR00126 | TCCACTCTTGACCAATTTTGAG | CACAAGAGGAAGCTATCGCA |
| 6 | SSR18956 | CGTATGTACGACAAAATGTGAACAG | TCGAAACCTCAATACTTCTACCAA |
| 6 | SSR02021 | TAAACATGGCTTCCTCCTCC | CTCTCTTTTCTCACACCCACAG |
| 6 | SSR23037 | GGGTAATGATCCAAATACCAAA | CCTTCCCTCATCTGTTGTTTT |
| 6 | SSR22275 | ATACTTCGCCATTGACGCTT | TTACTAGGCGTTGTGGGAGC |
| 6 | SSR01148 | CGGAGAAAGGCTCAGAAACA | TGCACGCACATAAACTAGGG |
| 6 | SSR01012 | TCCAAAAATCGCGACCTAAA | GTGAGCCGTTGATTTCTCGT |
| 6 | SSR13251 | GGTCAATCCAAAAGAGAAAGCA | ATCAACACCATTGACGACCA |
| 6 | SSR03962 | ATGGAGCCCTAATCACGTTG | CCGGCCAAACCCTATAAGAG |
| 6 | SSR23109 | AGTGGCTCCATTGTTGGAAG | CCTGCAAAATCAAGACCGTT |
| 6 | SSR10476 | ACAATGTTGAGTGGGTGGTT | TGGTTCACTGATGATGACCTG |
| 6 | SSR19174 | TGCCGTACACAAATTTCAAATAC | GAAGTATAATATGCACAAATCCCA |
| 6 | C80 | TTGATTTGAGTGTTTGAAATTGAG | ATAGCTTCGTTGGCATTGACATT |
| 6 | SSR30353 | GGACCATTAACTTCCACCCC | ATGGGATTTGCCTGTAATGC |
| 6 | SSR12283 | GACAATTGATGTCCCATTACCA | GCAGCAAGAATCATGTCACC |
| 6 | SSR07513 | GGAGCCATTGAAGAACAAGC | AAAAGGTCCAACCCAAAACC |
| 6 | SSR15238 | TGGGAGACAATTTATCAGTCCA | TGGTCTTCCTTATGCAAGCTCT |
| 6 | SSR13295 | AAATCCCCTCACCTCATTCC | TCAATTAAAGTAGACGTTTCGAATAA |
| 6 | SSR10829 | TGTAATGCCACGTCACACCT | AAGCCAAAGGGGTTTGAAAT |
| 6 | SSR03527 | TTGAGCACTTTGAATTATGGGA | GGCCAACCTTTGTTGTCAAT |
| 6 | SSR14061 | CCCAAGTTCTTGTGTTCTATGC | TGCACGTGATTTGGTTTTGT |
| 6 | UW083909 | TGAGGTGAAGCGTGAGAAGA | TTCAACTCATTATGGATTGGATT |
| 6 | SSR16710 | TTGGAGAGTGTGGTTTCTGTT | AAAATGTTGATTGGAAGAGCC |
| 6 | UW084119 | GGTGGGACCTTCTACTCTTT | CAAAATTCTCATCCTCAACC |
| 6 | SSR16882 | CACCTCAACTCCTCCATTCAA | TGGAGGTCATTGAGACTTGCT |
| 7 | SSR18648 | GCCATGAATGGAAACATGAA | TAAATTTTCCCAAATCCCCC |
| 7 | SSR00931 | GGACATCCCCATTGAATTTG | ACGTGGGGTCGACGTAATAA |
| 7 | SSR11742 | GCTATCCCCAAGGATGATGA | AGCTTGGCTTCGTCTTTTGA |
| 7 | SSR22777 | CTTCTCTCGGCCCAGTACAC | TTAATGTCCCACACTTGGCA |
| 7 | UW085407 | GAGCTTTTGGGAAAAGTCA | AGAAGATGGGGCCAGAATTT |
| 7 | SSR12994 | TCATCCTGCAATGCAAAGAC | ACCGTGGATCTTAAGCCAGA |
| 7 | SSR16001 | GCCTGTTTTCGGGCTAAACT | TCCAACTAATTTAATTTTGGGCTT |
| 7 | SSR13188 | GGGAATTTTCATTTATTTGGTCA | GGGAATTGGGATCCCTACAA |
| 7 | m44104 | TTTCCCGCATTGATTTTCTC | GAGAAACGCTTCCCACAAAC |
| 7 | SSR00926 | TAAAAGTAGCCGTTGGGTGC | TGACTTTCTGCACCTCTTTATCA |
| 7 | SSR22097 | ACGGTCATCCGAATTCTCAG | CAACAAACGATCCAACATCG |
| 7 | SSR00688 | AAGCCACCTTCCCTCTCAAT | GCAAACTGTGGGACGTTTCT |
| 7 | SSR01898 | AAAAATGTCAGAGAAGTGGATTGA | TTTGTGTTAGAGAAAGAGAGAGAGAG |
| 7 | SSR20583 | AACGTAATCGAAGGGTTGGA | CGCTTTAATTTTCAATGGGC |
| 7 | UW084365 | CAATTTGGGACTTCAAAGTCAGT | CGATGGGTTCAATAGATCTTGATAG |
| 7 | UW084390 | TGGGAAACAACTAACATACGTG | TCCATTTTCTTAATTTCGTTATTTGA |
| 7 | SSR21936 | TTGGTTGGAAAAAGGAAGGTT | GGGCAGAGGCTTTTTCAATA |
| 7 | UW083994 | CCACTCAGCAAGACATGGAA | TGCAAAATGTAAATTTGTGAAACTG |
| 7 | SSR30647 | GAAATGAGAGCAAGTTGCAAAA | AGGCGTAAATCTGACCGTTG |
